# A simple and automated method for ^161^Tb purification and ICP-MS analysis of ^161^Tb

**DOI:** 10.1186/s41181-022-00183-y

**Published:** 2022-12-02

**Authors:** Scott W. McNeil, Michiel Van de Voorde, Chengcheng Zhang, Maarten Ooms, François Bénard, Valery Radchenko, Hua Yang

**Affiliations:** 1 Life Sciences Division, TRIUMF, 4004 Wesbrook Mall, Vancouver, BC V6T 2A3 Canada; 2 NURA Research Group, Belgian Nuclear Research Center (SCK CEN), Boeretang 200, 2400 Mol, Belgium; 3 Department of Molecular Oncology, British Columbia Cancer Research Institute, 675 West 10th Ave., Vancouver, BC V5Z 1L3 Canada; 4 Department of Chemistry, University of British Columbia, 2036 Main Mall, Vancouver, BC V6T 1Z1 Canada; 5 Department of Chemistry, Simon Fraser University, 8888 University Dr, Burnaby, BC V5A 1S6 Canada

**Keywords:** ^161^Tb, Radionuclide purification, Automation, ICP-MS

## Abstract

**Background:**

^161^Tb is a radiolanthanide with the potential to replace ^177^Lu in targeted radionuclide therapy. ^161^Tb is produced via the neutron irradiation of [^160^Gd]Gd_2_O_3_ targets, and must be purified from ^160^Gd and the decay product ^161^Dy prior to use. Established purification methods require complex conditions or high-pressure ion chromatography (HPIC) which are inconvenient to introduce in a broad user community. This study aims to find a simpler small solid-phase extraction (SPE) column method for ^161^Tb purification that is more suitable for automation with commercially available systems like TRASIS.

**Results:**

We first tested the distribution coefficients on TK211 and TK212 resins for the separation of Gd, Tb, and Dy, and subsequently developed a method to separate these metal ions, with an additional TK221 resin to concentrate the final product. A side-by-side comparison of the products purified using this new method with the HPIC method was undertaken, assessing the radionuclidic purity, chemical purity regarding Gd and Dy, and labeling efficiency with a standard chelate (DOTA) and a novel chelate (crown). The two methods have comparable radionuclidic purity and labeling efficiency. The small SPE column method reduced Gd content to nanogram level, although still higher than the HPIC method. An ICP-MS method to quantify ^161^Tb, ^159^Tb, ^160^Gd, and ^161^Dy was developed with the application of mass-shift by ammonia gas. Last, ^161^Tb produced from the small SPE column method was used to assess the biodistribution of [^161^Tb]Tb-crown-αMSH, and the results were comparable to the HPIC produced ^161^Tb.

**Conclusions:**

^161^Tb was successfully purified by a semi-automated TRASIS system using a combination of TrisKem extraction resins. The resulting product performed well in radiolabelling and in vivo experiments. However, improvement can be made in the form of further reduction of ^160^Gd target material in the final product. An ICP-MS method to analyze the radioactive product was developed. Combined with gamma spectroscopy, this method allows the purity of ^161^Tb being assessed before the decay of the product, providing a useful tool for quality control.

**Supplementary Information:**

The online version contains supplementary material available at 10.1186/s41181-022-00183-y.

## Background

In recent years, there has been an increase of interest in new generation radionuclides with potential use in cancer therapy or imaging. Terbium (Tb) isotopes stand out by having great potential to perform on multiple fronts of cancer therapy/diagnostics (Müller et al. 2012). There are four medically relevant Tb isotopes identified: ^149^Tb for alpha therapy and positron emission tomography (PET) imaging (Müller et al. 2016), ^152^Tb for PET imaging (Baum et al. 2017), ^155^Tb for single-photon emission computerized tomography (SPECT) imaging (Favaretto et al. 2021) and ^161^Tb for β^−^/Meitner-Auger (MA) therapy and SPECT imaging, covering all major nuclear medicine modalities. Among the Tb isotopes, ^161^Tb has drawn a lot of attention because it is a β^−^ and MA electron emitter with suitable half-life (t_1/2_ = 6.96 d, Eβ^−^_av_ = 154 keV ~ 12.4 e^−^, 46.5 keV per decay) (Colins et al. 2022), can be produced at clinical quantities, and can potentially work in tandem with the SPECT imaging radionuclide ^155^Tb. ^161^Tb displays similar chemical behaviour and half-life to ^177^Lu (t_1/2_ = 6.65 d, Eβ^−^_av_ = 134 keV) while exhibiting more potent radiotherapeutic properties due to additional MA and conversion electrons emissions, especially for the treatment of multiple metastases (Lehenberger et al. 2011) (Bernhardt et al. 2021). Preclinical studies have directly compared the tumour treatment efficacy of [^161^Tb]Tb-cm09 (folate conjugate) (Müller et al. 2014), [^161^Tb]Tb-PSMA-617 (Müller et al. 2019) and [^161^Tb]Tb-DOTA-chCE7 (anti-L1CAM mAb) (Grünberg et al. 2014) to their ^177^Lu counterparts, and the results show evidence of the superior therapeutic efficacy of ^161^Tb. In 2021, the first in-human feasibility study with [^161^Tb]Tb-DOTA-TOC was reported, marking a new era for clinical use for this radionuclide (Baum et al. 2021).

**Fig. 1:**
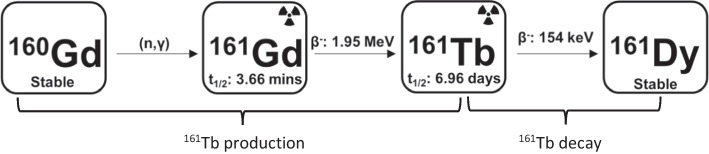
Production and decay of ^161^Tb

^161^Tb is produced via the neutron irradiation of enriched [^160^Gd]Gd_2_O_3_ targets and decays into stable ^161^Dy (Fig. 1) (Lehenberger et al. 2011). The challenge of the purification is to separate three neighbouring lanthanides gadolinium (Gd), terbium (Tb), and dysprosium (Dy). Several methods have been developed including those by Lehenberger et al. and Gracheva et al., typically involving a large cation exchange column for purification and a small secondary solid phase extraction (SPE) column for concentration of the final product (Lehenberger et al. 2011) (Gracheva et al. 2019). Although such methods achieve a high radionuclidic purity, to accommodate the complex elution systems, they require custom made modules and acid-resistent high performance ion chromatography (HPIC) systems that are not easily adaptable to different laboratories (Cassells et al. 2021). This paper reports a new method involving three small SPE columns with simple and predictable elution conditions, practical for smaller centres and lab settings. This method was semi-automated on a commercial module (TRASIS Mini AIO), and achieved purification efficiency comparable to the existing methods. Notably, the small SPE column method reduced Gd to nanogram level, although higher compared to HPIC method. During the development of this method, the use of  inductively coupled plasma mass spectrometry (ICP-MS) to analyse the radioactive product was also investigated. This was aided by an additional mass-shift using ammonia gas; ^161^Tb, ^159^Tb, ^160^Gd, and ^161^Dy were quantified. Together with gamma spectroscopy, one can obtain the impurity profile for both stable and radioactive metals without waiting for the material to decay. The automation process and quality control method developed can potentially faciliate the good manufacturing practice (GMP) production of ^161^Tb for clinical translation of this promising radionulide. Furthermore, we compared the labeling efficiency of ^161^Tb produced by small SPE column method with HPIC produced ^161^Tb, using a standard chelator (DOTA) and a novel chelator (crown), and both ^161^Tb sources showed similar labeling efficiency at various chelator concentrations. When used to prepare [^161^Tb]Tb-crown-αMSH, a melanocortin 1 receptor (MC1R) targeting radiopharmaceutical, HPIC produced ^161^Tb showed higher molar activity, consistent with its lower Gd content. In vivo evaluation of [^161^Tb]Tb-crown-αMSH using the ^161^Tb purified from both methods were conducted and showed similar biodistribution profiles in tumour bearing mice at 2 h post-injection, demonstrating the bioequivalence and preclinical use of ^161^Tb purified using the small SPE column method.

## Results

### Distribution coefficients (K_d_) measurements

**Fig. 2:**
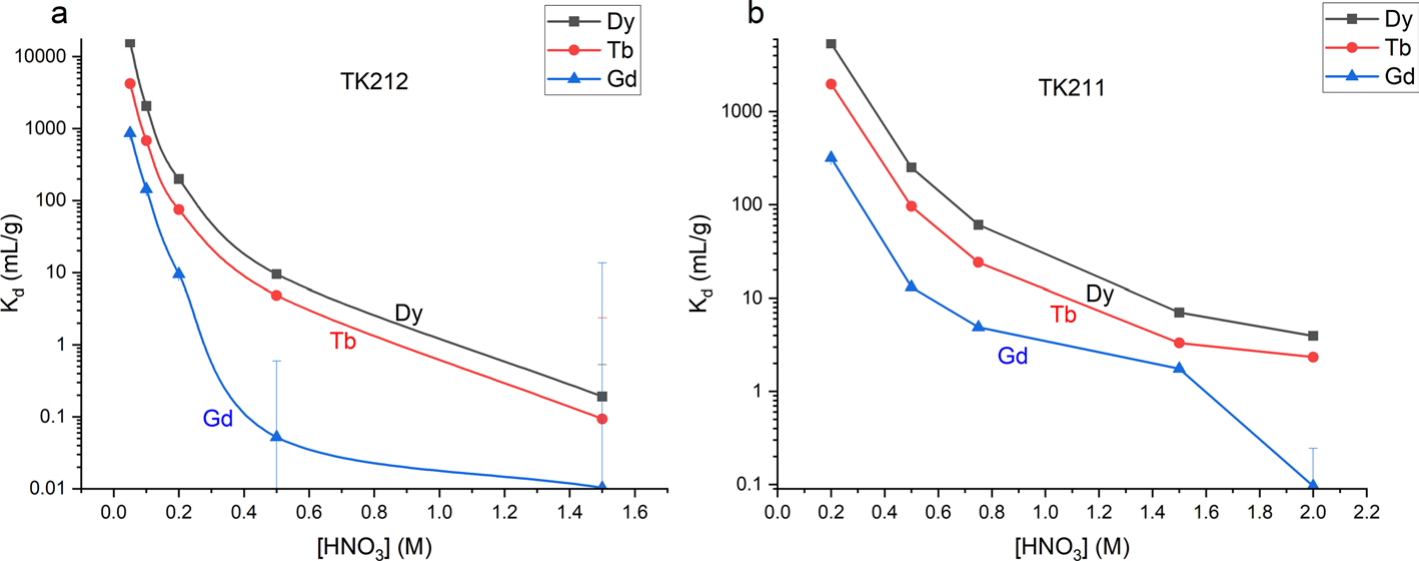
K_d_ plots for TK212 (**a**) and TK211 (**b**) resins measured at various HNO_3_ concentrations

The new TK212 and TK211 resins were evaluated for separation of the lanthanides. These resins are similar to LN resins but use mixed organophosphoric, organophosphonic and organophosphinic acid extractants that may work in synergy to improve selectivity. The solid support contains aromatic groups and the organic phase is mixed with small amounts of long chain alcohols. Such changes are said to make the resins more resisitant to radiolysis (Happel 2022). The distribution coefficients (K_d_) of Gd, Tb, and Dy on TK212 and TK211 resins in various concentrations of HNO_3_ were determined using ICP-MS (Fig. 2). Both resins have higher affinity to the lanthanides at lower HNO_3_ concentrations. TK212 has high K_d_ values for Tb below 0.2 M HNO_3_ while at the same acid concetration Gd is not retained by the resin, which allows Tb product to be extracted and the bulk of the Gd target matrix to be removed. TK211 can further remove trace Gd and separate Tb from Dy.

### Separation of non-radioactive Gd, Tb and Dy

**Fig. 3:**
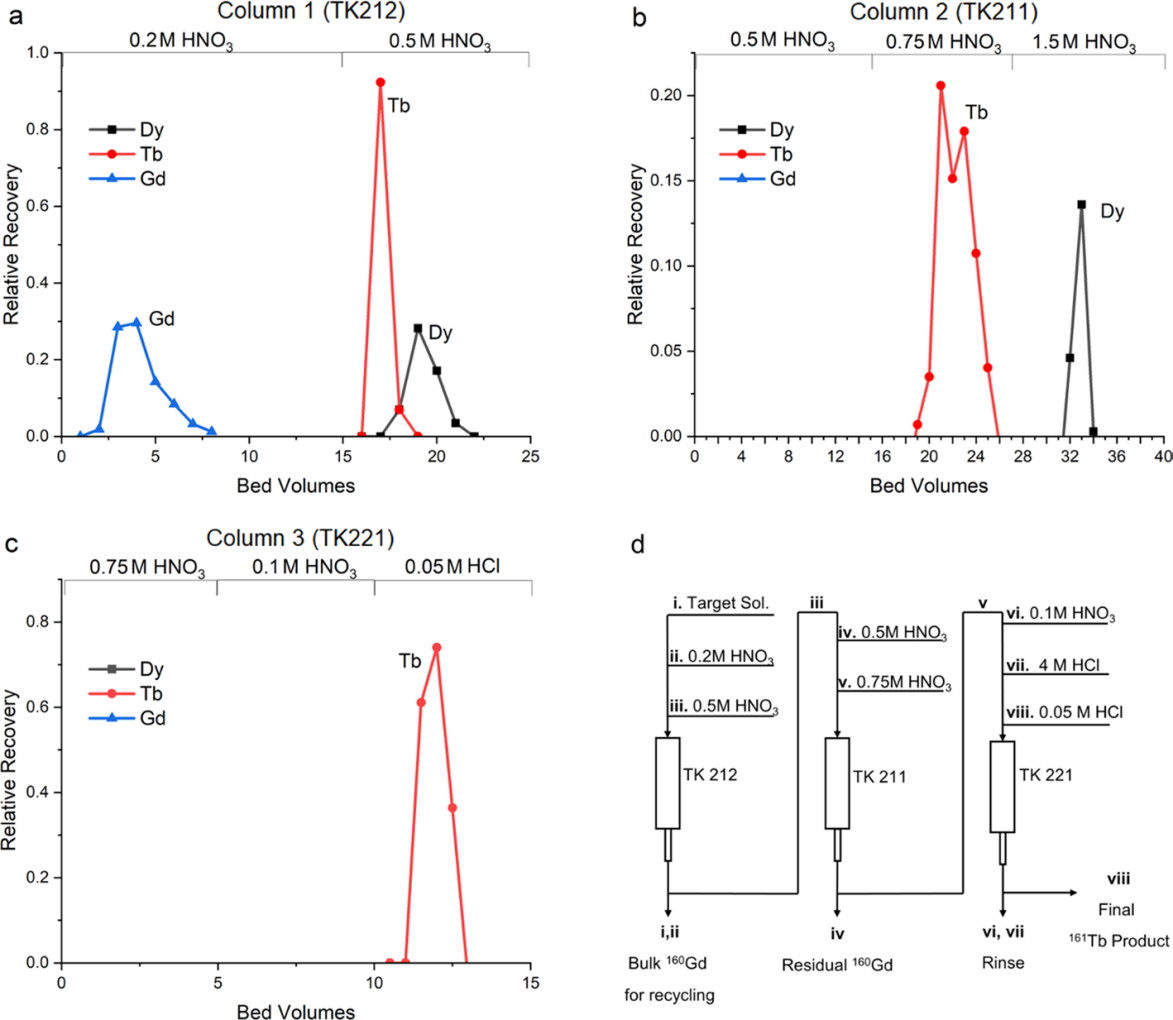
Elution profile of Gd, Tb, and Dy (10 mg, 1 mg, and 1 mg respectively) TK212 (**a**), TK211(**b**) and TK221 (**c**). Tb containing fractions were combined for loading on the next column to simulate purfication run. Column bed volumes were 1 mL for each column during cold test but were later optimized for active runs. Overall process diagram (**d**) note steps i–v can be performed automactically, while steps vi–viii must be performed manually

A third column is required to concentrate the final product and reduce the acid content so the product can be used for radiolabeling, a TK221 column was therefore introduced. The separation of natural Gd, Tb, and Dy was performed using three columns TK212, TK211, and TK221. Gd(NO_3_)_3_, TbCl_3_, and Dy(NO_3_)_3_ were used for the experiments and monitored with colormetric measurements using Arsenazo III reagent (Rohwer et al. 1995). 10 mg Gd(NO_3_)_3_, 1 mg TbCl_3_, and 1 mg Dy(NO_3_)_3_ were each dissolved in 100 µL 0.2 M HNO_3_ and loaded onto a 1 mL TK212 column. Most Gd was removed with 15 mL 0.2 M HNO_3_ wash (Fig. 3). Tb and Dy were eluted with 10 mL 0.5 M HNO_3_ and loaded directly onto a 1 mL TK211 column, which was rinsed with 15 mL 0.5 M HNO_3_ to remove any residual Gd. Tb was then eluted with 10 mL 0.75 M HNO_3_, whereas Dy can remain on the TK211 column or be eluted with 1.5 M HNO_3_. Finally, the third column (TK221, 1 mL) was used to concentrate Tb. Tb eluted from TK211 by 0.75 M HNO_3_ was loaded directly onto the TK221 column. This column was washed with 10 mL 0.1 M HNO_3_ and the final product was eluted with 5 mL 0.05 M HCl. It was later found that rinsing with 4 M HCl before eluting with 0.05 M HCl allowed for the Tb to be eluted as a sharper peak.

### Automation

**Fig. 4:**
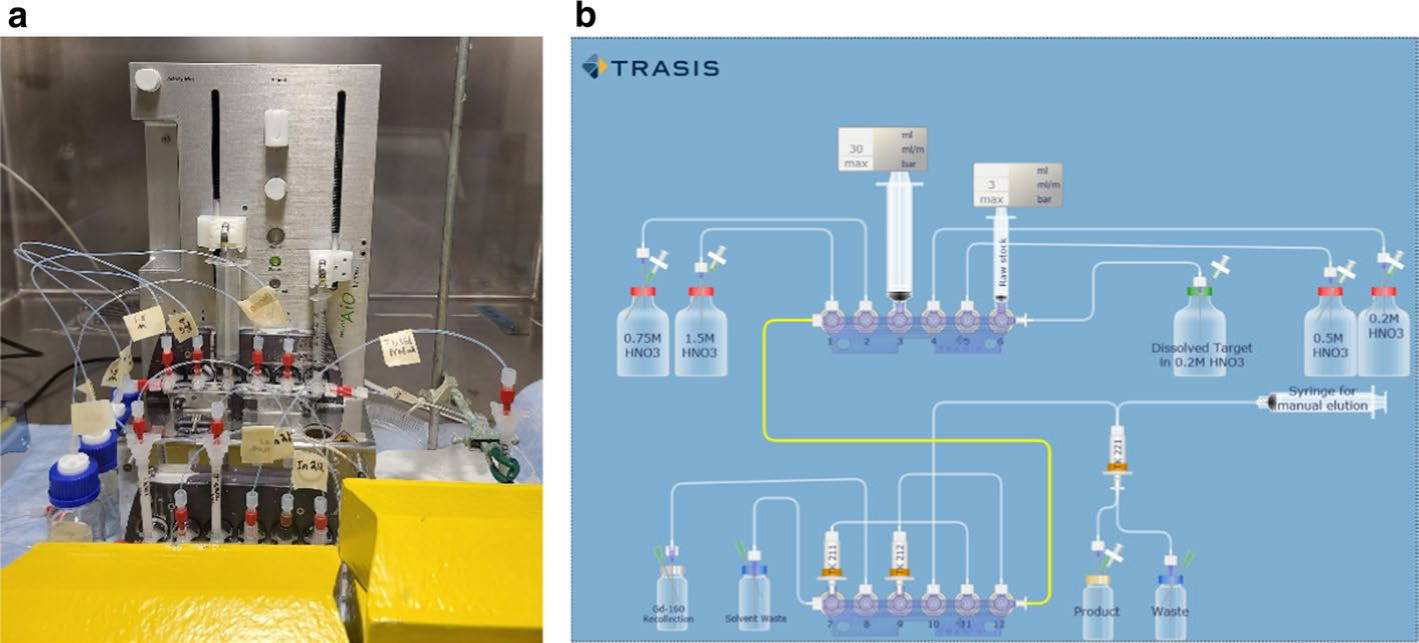
TRASIS Mini AIO module and components set-up within hotcell (**a**), TRASIS layout diagram (**b**)

The process outlined in Fig. 3d was automated using a TRASIS Mini AiO module (Fig. 4). The disposable cartridge is an advantage to prevent contamination between batches. Limited by the port numbers, the last column was eluted manually. Total separation time was 90 min with the semi-automated method, and 95% of the ^160^Gd was recovered in the recovery fraction as measured by ICP-MS.

### ^161^Tb purification

^161^Tb was produced at BR2 reactor (Mol, Belgium) from neutron irradiation of [^160^Gd]Gd_2_O_3_ targets. This material was dissolved in 1 M HNO_3_ and split into two portions. One portion was purified using the HPIC method (Cassells et al. 2021) at the Belgian Nuclear Research Center (SCK CEN). The other portion was added to H_2_O (final HNO_3_ concentration 0.08 M) and left unpurified. Three batches of activities were shipped to TRIUMF (Vancouver, Canada) and used for developing the small SPE column purification methods and subsequent radiochemistry and biology studies.

The unpurified ^161^Tb was purifed using the above described on a TRASIS module at TRIUMF (Fig. 3d), with the only change being the size of the third column TK221. The optimal size of the TK221 column was found to be much smaller at 30 µL. Such bed volumes allowed for the final ^161^Tb product to be sufficiently pure and sufficiently concentrated without the need for any evaporation from up to 10 mg of target material. To allow sufficient interaction with the liquid passing through, the 30 µL TK221 resin was packed in a 200 µL pipette tip with polyethylene frits cut in-house.

Semi-automated purification was performed three times using 50–110 MBq of activity from a single batch of ^161^Tb. The product was eluted with 0.05 M HCl (0.164–0.763 MBq/µL at end of synthesis (EOS)). The products were compared to the HPIC purfied ^161^Tb (2.78 MBq/µL in 0.05 M HCl at EOS) from the same batch. Results are summarized in Tables 1 and 2. The radionuclidic purity was measured by gamma spectroscopy after ^161^Tb has decayed (70 days) (Table 1). The new method removed the radioactive impurities in similar efficiency as the previously reported HPIC method. ^153^Gd, ^152^Eu, ^156^Eu, ^169^Yb, and ^192^Ir were not detected after purification.

**Table 1:** Summary of Radionuclidic purity decay corrected to EOS (ND = not detected)

	% Activity							
Sample	^161^Tb	^160^Tb	^153^Gd	^152^Eu	^156^Eu	^192^Ir	^169^Yb	All others*
Unpurified	99.9540	0.0063	0.0224	0.0030	0.0053	0.0017	0.0047	0.0020
HPIC purified	99.9947	0.0053	ND	ND	ND	ND	ND	ND
Small column purified	99.9939	0.0061	ND	ND	ND	ND	ND	ND

*All others include ^46^Sc, ^141^Ce, ^154^Eu and ^155^Eu. N = 1 for each sample. For small column purification, the product from 110 MBq purification was used

**Table 2:** Comparison of semi-automated small SPE column product to HPIC product^[a]^

	Mass (ng) [% of Total Mass^[b]^]				Average ^161^Tb Recovery (%)
Method	^161^Tb^[c]^	^159^Tb^[c]^	^160^Gd	^161^Dy	
HPIC (n = 1)	17.5 ng [91.7%]	1.4 ng [7.2%]	0.2 ng [0.9%]	< 0.1 ng [< 0.2%]	NA
Semi-Automated Small Column (n = 3)	17.5 ng [45.5 ± 3.9%]	6.1 ± 1.3 ng [15.8 ± 3.5%]	13.2 ± 3.5 ng [34.4 ± 9.2%]	1.6 ± 2.8 ng [4.3 ± 7.4%]	77 ± 13

^[a]^Semi-automated small column purification was performed on three portions (50 MBq, 50 MBq, and 110 MBq) split from a single batch and the results expressed as average ± standard deviation. HPIC purification was performed with the same batch material. All results decay corrected to EOS from each purification and normalized to 75 MBq ^161^Tb. All samples measured by ICP-MS. ^[b]^Total Mass defined as the sum of (^161^Tb + ^159^Tb + ^160^Gd + ^161^Dy). ^[c]^The small column purified ^161^Tb was purified > 1 week after the HPIC purified thus accounting for the lower ^161^Tb/^159^Tb ratio

**Fig. 5:**
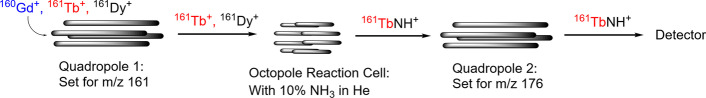
Resolution of ^161^Tb and ^161^Dy ions via NH_3_ Mass Shift mode with ICP-MS/MS

To quantify ^160^Gd (target material), ^159^Tb (natual Tb), and ^161^Dy (decay product) along with ^161^Tb, an ICP-MS method was developed. On typical ICP-MS instruments, ^161^Dy and ^161^Tb would not be differentiable as the resolving power required of the machine would be too high. In some cases, Triple Quad reaction cell equipped instruments are able to use differences in chemical reactivity to differentiate between isobars of different elements. In the case of Tb and Dy, previous work has revealed that ammonia gas could be used to preferentially shift Tb’s mass in the result of TbNH^+^ (M + 15) product ion (Fig. 5) (Sugiyama & Nakano 2022). Since Dy does not react sufficiently with with NH_3_, the use of NH_3_ mass shift mode can allow one to separate and quantify ^161^Tb, without significant interference from ^161^Dy. This method is validated using non-radioactive Tb and Dy.

Due to this observed reactivity ^161^Tb was quantified by first measuring the counts per second obtained while analyzing quadrupole 1 at m/z 161 and quadrupole 2 at m/z 176, with the reaction cell in NH_3_ mode. This result could then be quantified using a calibration curve generated with natural ^159^Tb subjected to the NH_3_ tune mode (Additional file 1). With the concentration of ^161^Tb established, ^161^Dy could be quantified by subtracting the calculated signal resultant of ^161^Tb from the He mode value of m/z 161 and then applying the new value to a calibration curve for ^161^Dy. It should be noted since ^160^Gd and ^160^Dy are natural isobars, the use of NH_3_ mass shift mode is required to accurately quantify ^160^Gd present in samples as well by ensuring interference for Dy isotopes is removed. In this way ICP-MS technology allowed for the real time quantification of ^160^Gd, ^161^Tb, and ^161^Dy in purified samples, and it was not necessary to let the ^161^Tb decay prior to measuring.

The results indicate that using the semi-automated method the amount of Gd is reduced from 1.5 mg to trace level (13.2 ± 3.5 ng, n = 3) (Table 2), accounting for 34.4 ± 9.2% of the total mass (decay corrected to EOS), while the HPIC method almost completely removed ^160^Gd (< 1%). No other stable metal impurities of significant quantity were detected in the samples from either method. As a result, the total % mass for ^161^Tb produced from the small columns is 45.5 ± 3.9%. Considering the impurity mass is at nanogram level, the purification process is efficient and the product is suitable for radiolabeling experiments.

We also tried performing purification entirely manually, and the results show better overall removal of Gd (2.3 ng for 75 MBq ^161^Tb). This may be due to the lack of dead volume when performed manually, although further invetigation is required. Subsequent labeling and animal studies were performed using the ^161^Tb purified semi-automatically.

An alternative ICP-MS product ion was also examined later in the study. The two different mass shift reactions investigated for ^161^Tb quantification were Tb^+^→TbNH^+^ (M + 15) and Tb^+^→TbNH(NH_3_)^+^ (M + 32). As seen in the results in Table 3, the M + 15 tuning mode gave an average difference of 14.9% when compared to gamma spectroscopy and typically over reported the ^161^Tb content. Although the M + 32 tuning mode resulted in a more accurate result with an average error of 3.3%, the sensitivity of the M + 15 mode was found to be higher than that of the M + 32 mode, 21 counts per second/ppt and 12 counts per second/ppt, respectively.

**Table 3:** Comparison of ICP-MS calculated ^161^Tb activity to HPGe Gamma spectroscopy measured ^161^Tb activity

Trial	Activity gamma spectroscopy (MBq)	Activity ICP-MS (MBq)		% Difference	
		Tb^+^→TbNH^+^ (M + 15)	Tb^+^→TbNH(NH_3_)^+^ (M + 32)	% Difference (M + 15)	% Difference (M + 32)
1	108.1	129.4	106.3	19.7	1.6
2	55.2	64.7	56.6	17.1	2.6
3	737.9	795.6	695.6	7.8	5.7
Average				14.9	3.3

% Difference was calculated as the absolute difference between ICP-MS calculated activity and gamma spectroscopy measured activity over gamma spectroscopy measured activity

### Radiolabeling experiments

**Fig. 6:**
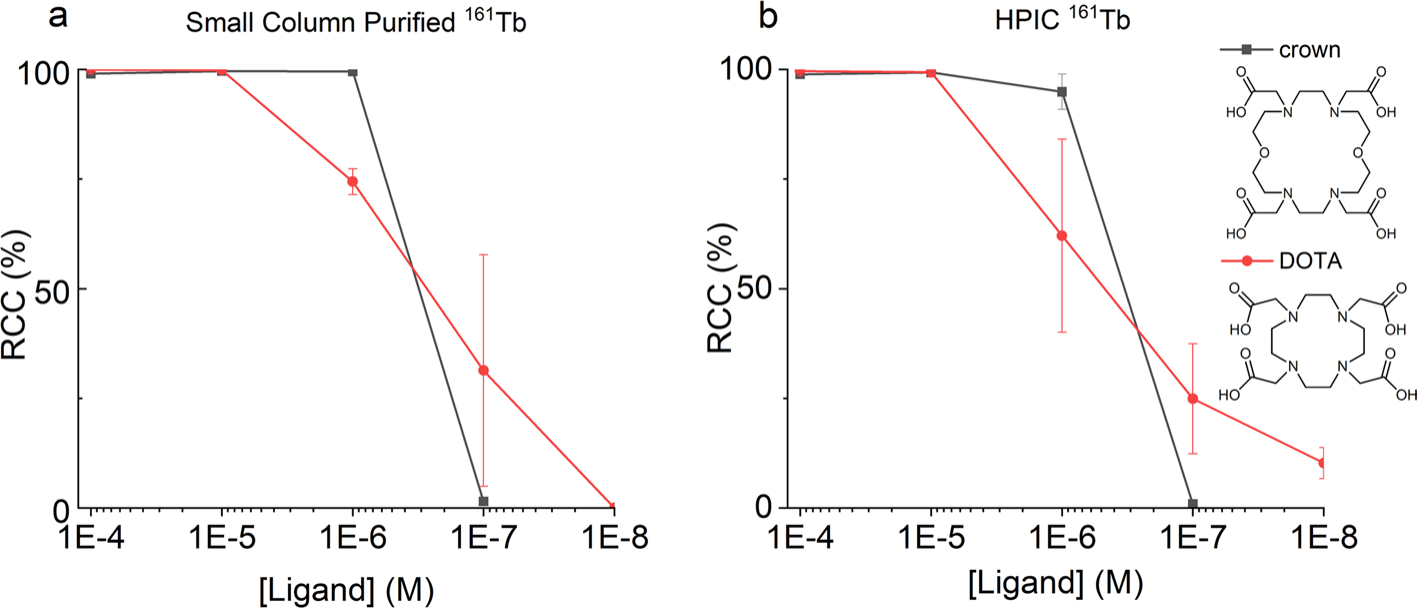
Labelling comparison between small column (**a**) and HPIC (**b**) purified ^161^Tb (100 kBq for each reaction) with crown (room temperature, 30 min), and DOTA (85 °C, 30 min). RCC = radiochemical conversion

The radiolabeling of each ^161^Tb product was compared using two chelators (DOTA and crown), using both the ^161^Tb produced from HPIC and from small SPE columns. Crown is a new macrocyclic chelator developed in prior studies in our group and is capable of coordinating actinium (Ac^3+^) efficiently at room temperature (Yang et al. 2020). It has recently been discovered that crown can also label Tb^3+^ at room temperature (Wharton et al. 2022). With decreasing concentrations of the chelators, the critical chelator concentration required for high efficiency labeling was determined (Fig. 6). HPIC purified ^161^Tb and small column purified ^161^Tb (semi-automated) performed similarly in these experiments. For DOTA, ^161^Tb from both methods labeled quantitatively at 10^–5^ M, and the radiochemical conversion (RCC) went down gradualy from 10^–6^ to 10^–7^ M. For crown, ^161^Tb from both methods achieved quantitative labeling at 10^–6^ M, and the RCC dropped to 0 at 10^–7^ M.

### Highest apparent molar activity determination

The highest apparent molar activity achievable for [^161^Tb]Tb-crown-αMSH was determined for the ^161^Tb purified via HPIC and small column purification. It was done by addition of increasing amounts of ^161^Tb activity (7.1 – 14.6 MBq) to a constant amount of crown-αMSH (0.1 nmol), with the highest molar activity being defined as the largest amount of activity to still achieve quantitative RCC to the [^161^Tb]Tb-crown- αMSH product. Using the ^161^Tb purified by small columns and TRASIS module, [^161^Tb]Tb-crown-αMSH at 85.4 MBq/nmol was prepared, while with HPIC purified ^161^Tb, 144.9 MBq/nmol was achieved (Table 4). The lower apparent molar activity achieved is in agreement with the lower ^161^Tb purity for semi-automated small column purifed ^161^Tb (45.5% vs. 91.7%) caused mainly by the higher Gd content.

**Table 4:** Comparison of highest achievable apparent molar activity for [^161^Tb]Tb-crown-αMSH synthesized from both the small column and HPIC purified ^161^Tb

Source of ^161^Tb	Apparent molar activity* (MBq/nmol)
Small column (semi-automated)	85.4
HPIC	144.9

*Apparent molar activity = activity for quantitative labeling (MBq) / amount of crown-αMSH in reaction (nmol)

### In vivo biodistribution study

**Fig. 7:**
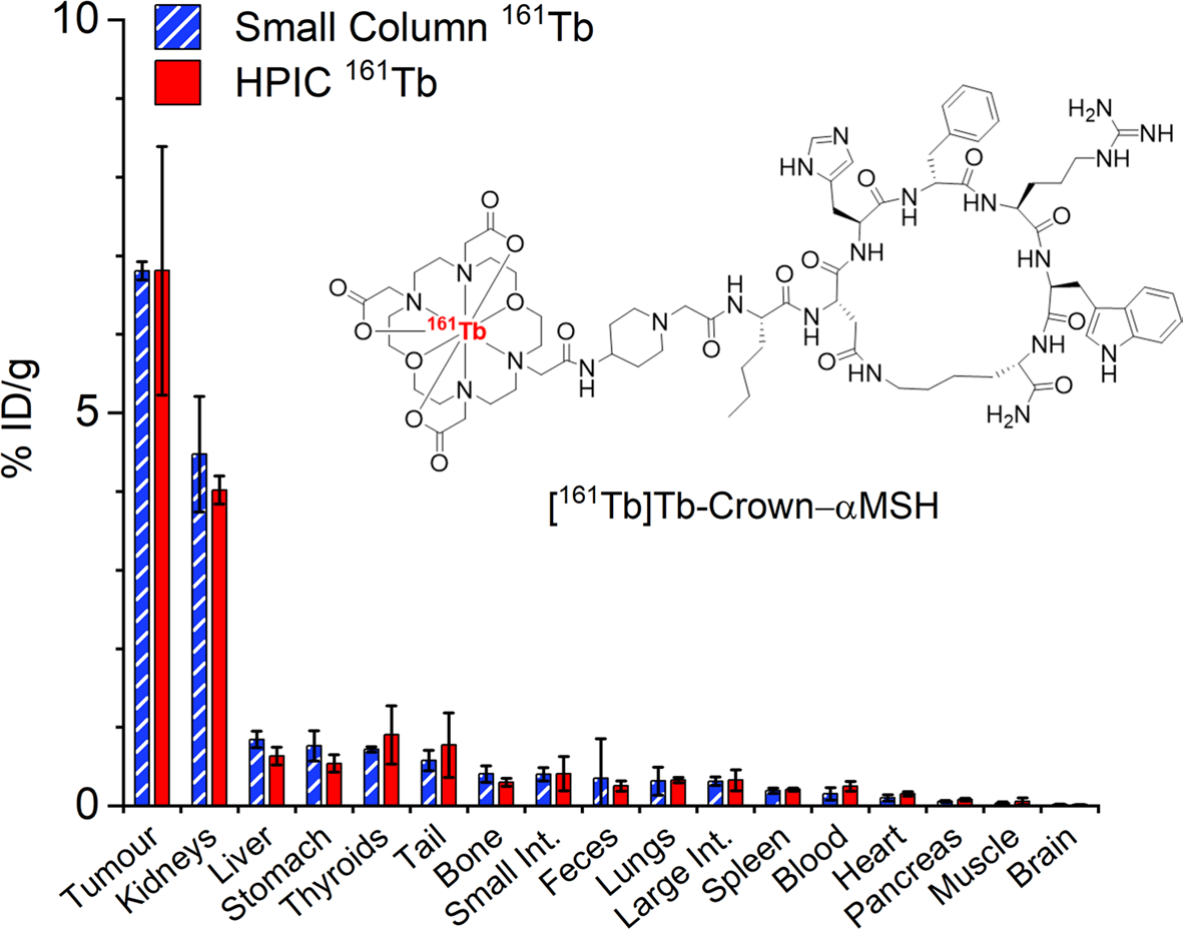
Biodistribution of [^161^Tb]Tb-crown-αMSH in male C57BL/6 J mice bearing B16-F10 tumour at 2 h post injection. Error bars reflect standard deviation (n = 3)

To investigate whether the ^161^Tb purified from HPIC and small columns are comparable in vivo, [^161^Tb]Tb-crown-αMSH was synthesized using ^161^Tb from each of these two methods and each radiotracer was assesssed in a biodistribution study in mice bearing B16-F10 tumours at 2 h post injection. (Fig. 7). αMSH is a cyclic peptide targeting MC1R, which is expressed specifically in melanomas. The expression of MC1R in normal tissues and organs is very low, making it an interesting target for developing imaging or therapeutic radiopharmaceuticals. Due to the relatively low receptor density of MC1R in melanoma, high molar activity and low injected peptide mass (< 50 pmol per animal) is required for radiopharmaceuticals targeting this receptor to have a good tumour uptake without saturating (blocking) the receptors. Thus this is a good system to test the ^161^Tb produced from different methods. In this case, [^161^Tb]Tb-crown-αMSH using similar amounts of ^161^Tb purified from HPIC or semi-automated small columns showed almost identical biodistribution profiles (Fig. 7). The results demonstrated ^161^Tb purified from these two methods are interchangable for this preclinical evaluation.

## Discussion

Herein a simple method to purify ^161^Tb was developed. The use of small SPE columns in this method greatly reduces the amount of resin required (13 mL for reported HPIC method, 2 × 1 mL resin for small SPE column method). Compared to acid-resistant HPIC purification, this method is likely to prove more cost efficient and easier to operate, thus suitable for smaller centers and labs.

The elution of the nuclides of interest (^160^Gd, ^161^Tb, and ^161^Dy) are dependent on the concentration of HNO_3_ used, making it easy to predict which fractions to collect, therefore easily adaptable for automation. Semi-automation was demonstrated on a commercial TRASIS module, and full automation can be easily achieved by using a system with more manifolds and solvent interchanges.

^161^Tb produced from this new method has a similar radionuclidic purity compared to the reported HPIC method. The chemical purity studies showed the presence of ^160^Gd at nanogram level, in similar proportion to the amount of ^161^Tb, and this Gd level is higher than the HPIC method. Increasing the amount of resins used in the first two columns (TK212 and TK211) to 1.5 mL or 2 mL may improve the removal of Gd and will be investigated. In the future, a resin with reversed elution sequence (Dy, Tb, Gd) will be incorporated as an effort to trap Gd from the final product. Column dimesions, eluent flow rate, and ways to reduce the dead volumes will also be further investigated.

An ICP-MS method to analyze ^161^Tb, ^160^Gd, ^161^Dy was developed. This method used NH_3_ gas to shift the mass of ^161^Tb ions (^161^Tb^+^→^161^TbNH^+^) to a different mass (M + 15) and eliminate interference from ^161^Dy, thus allowing the chemical purity of the final product to be analyzed before decay. Upon comparing different NH_3_ mass shift modes it was found that the ^161^Tb^+^→^161^TbNH(NH_3_)^+^ (M + 32) provides a more accurate representation of ^161^Tb content when compared to gamma spectroscopy and thus this mode is recommended for future ICP-MS analysis of ^161^Tb. This method can potentially be useful for quality control in ^161^Tb manufacture.

^161^Tb produced by the small SPE column method showed similar labeling results with DOTA and crown chelators compared to ^161^Tb produced from HPIC method, and the highest achievable apparent molar activity for [^161^Tb]Tb-crown-αMSH is lower as expected. In vivo evaluation of [^161^Tb]Tb-crown-αMSH made with ^161^Tb purified from both methods displayed almost identical biodistribution of [^161^Tb]Tb-crown-αMSH in mice bearing B16-F10 tumours at 2 h post injection, demonstrating the ^161^Tb purified from small columns is interchangeable with HPIC purified ^161^Tb for such purposes. Another benefit to this method is the potential to extend the shelf life of ^161^Tb by removing any ^161^Dy that will acculmulate whether it be from prolonged shipping or simply radionuclidic decay. ^161^Dy is chemically simillar to ^161^Tb and thus any ^161^Dy present can affect radiolabelling. This method provides a convient way to remove grown in ^161^Dy.

## Conclusion

In summary, a simple and effective small SPE column based method of purifying ^161^Tb produced from ^160^Gd(n,γ)^161^Gd→^161^Tb reaction is reported. ^161^Tb purified using this new method is comparable to the ^161^Tb obtained by HPIC in terms of radionuclide purity and chemical purity, although a higher level of Gd (in the ng level) was observed. The new method also performed similarly in labeling tests and in vivo studies compared to HPIC method.

During the investigation of this new separation method, an ICP-MS method for analyzing radioactive ^161^Tb in the presence of ^159^Tb, ^160^Gd, and ^161^Dy was developed using the mass shift by ammonia gas. Combined with gamma spectroscopy, this ICP-MS method can give an impurity profile without the need for samples to decay, which is useful for the quality control of ^161^Tb.

Future work will focus on improving the removal of Gd by optimizing column parameters (mass, dimension, flow rate, etc.) or introducing a new resin that helps trap trace Gd. Full automation including a target dissolution unit using more complex commercial modules will be very useful for upscaling and handling more target material.

Overall, this simple new method is useful for purifying the promising ^161^Tb and other Tb isotopes in lab setting and small centers, or for extanding the shelf-life of ^161^Tb, and may inspire new separation methods for other radiolanthanides.

### Methods

#### Materials

Trace metal basis (> 99.99%) Gd(NO_3_)_3_·6H_2_O, TbCl_3_·6H_2_O and Dy(NO_3_)_3_ were purchased from Sigma-Aldrich. Trace metal basis (> 99.999%) concentrated HNO_3_ (70%) purified by redistillation was purchased from Sigma-Aldrich. Trace metal grade concentrated HCl was purchased from Fisher Scientific. Arsenazo III was purchased from Sigma-Aldrich. ICP-MS standard solution was purchased from Agilent. Milli-Q water was provided in-house. TK211, TK212, TK221 resins were provided by TrisKem. Silica plate on aluminum backing was purchased from Sigma-Aldrich and cut to 2 × 10 cm pieces. SG-iTLC plate was purchased from Agilent and cut to 2 × 12 cm. Gamma spectroscopy was collected using N-type co-axial high purity germanium (HPGe) gamma spectrometer (Canberra Industries) and the spectra were analyzed using the Genie 2000 software package (Version X, Canberra Industries). RadioTLC was scanned using an Eckert & Ziegler AR2000 TLC scanner equipped with P10 gas and then analyzed by WinScan software. RadioHPLC was carried out using an Agilent 1260 HPLC equiped with a GABI Star radioactive HPLC flow monitor.

#### K_d_ development procedure

Natural TbCl_3_, Gd(NO_3_)_3_, and Dy(NO_3_)_3_ (~ 1 mg/mL for each salt) were separately dissolved in nitric acid of varying concentrations. 1 mL of the metal-containing solution was then mixed with 100 mg of dry resin in centrifuge tubes, they were then allowed to equilibrate for 2.5 h on a tube-shaker with rapid stirring. After the equilibration time, the contents of the tubes were filtered via 0.22 µm PTFE syringe filters, and the filtrate was analyzed by ICP-MS. The metal concentration on the resin was determined by the difference between the metal concentration in the initial stock solution and the metal concentration in the equilibrated solution, similar to the work of Mastren et al. (2018) using the following formula:

$$K_{d} = \frac{{C_{resin} }}{{C_{aq} }} = \frac{{M_{T} - M_{aq} }}{{M_{aq} }}*\frac{V}{m}$$

where C_resin_ is the concentration of metal absorbed on the resin, C_aq_ is the concentration of metal in the aqueous portion, M_T_ is the total mass of metal added, M_aq_ is the mass of metal found in the aqueous portion, V is the volume of the aqueous portion in mL, m is the mass of the resin in g. The resulting formula expresses K_d_ as [M]_resin_/[M]_solution_ with units of mL/g.

#### Isolation procedure development

The isolation of Tb was conducted by first testing each column/resin individually with one metal at a time. Resins were preequilibrated in 20% aq. MeOH for 1 h to generate a slurry for optimal packing. 1 mL of each resin (TK212, TK211, or TK221) was packed into a 4 mL reservoir with polyethylene frit. Each column was rinsed with 10 bed volumes of HNO_3_ (0.2 M HNO_3_ for TK212, 0.5 M HNO_3_ for TK211 and 0.75 M HNO_3_ for TK221). Metal salt (10 mg Gd(NO_3_)_3_, 1 mg TbCl_3_, or 1 mg Dy(NO_3_)_3_) was each dissolved in 100 µL of 0.2 M HNO_3_ and then loaded to the columns individually. TK212 and TK211 columns were eluted with 0.2, 0.5, 0.75 or 1.5 M HNO_3_. TK221 column was eluted with 0.75 M HNO_3_, 0.1 M HNO_3_, or 0.05 M HCl.

Fractions of 1 bed volume (1 mL) were collected manually and then analyzed colorimetrically with Arsenazo III indicator. UV calibration curves were used to determine the amount of Gd, Tb, and Dy in each fraction. Due to the limitations of the Arsenazo III complex, only one metal could be tested at a time on the columns. Through several experiments optimal elution conditions were established. The revised conditions are as follows: The target solution is loaded onto TK212 in 1 mL of 0.2 M or lower HNO_3_, TK212 is then rinsed with 10 bed volumes of 0.2 M HNO_3_, this portion is collected for target recycling as it contains the bulk of the Gd. Next the Tb and Dy are eluted from the TK212 column with 10 bed volumes of 0.5 M HNO_3_, this portion is directly loaded onto the TK211 column. The TK211 column is then rinsed with 35 bed volumes of 0.5 M HNO_3_ to further reduce the Gd content. Next 15 bed volumes of 0.75 M HNO_3_ is used to selectively elute Tb off the TK211 column and leave the bulk of the Dy retained. This portion is directly loaded on to a TK221 column. The TK221 column is first rinsed with 5 bed volumes 0.1 M HNO_3_ before finally eluting with 6–10 bed volumes of 0.05 M HCl to obtain the final Tb product. The final step is fractionated to ensure a more concentrated Tb product.

#### Automation

The above-described procedure was automated using a TRASIS AIO Mini module. The module syringe pumps were used to load/ elute the metals onto TK212, TK211, and TK221 columns.

Once the terbium was isolated and loaded onto the TK221 column the column was disconnected from the automated system and manually rinsed with 0.1 M HNO_3_ followed by 4.0 M HCl then the Tb was eluted in a small volume of 0.05 M HCl.

#### ICP-MS analysis of non-radioactive samples

All ICP-MS measurements were performed using Agilent 8900 #100 Triple Quad instrument equipped with H_2_, He, O_2_, and 10% NH_3_ in He as cell gasses and an Agilent SPS-4 autosampler.

A 16 multielement standard (Agilent) containing Gd, Tb, and Dy was used to generate calibration curves for the ICP-MS analysis. Before all runs, the instrument was tuned using standard tuning parameters for no gas and Helium tune modes. Helium Tune mode was used for quantifications. All samples and standards were prepared gravimetrically, and all dilutions were carried out using ultra-pure 2% (w/w) HNO_3_. Measured nuclides were ^159^Tb, ^157^Gd, and ^163^Dy.

#### ^161^Tb production and purification

[^160^Gd]Gd_2_O_3_ targets were irradiated at BR2 reactor for 7 days using a high thermal neutron flux of 3 × 10^14^ neutrons/cm^2^/s. The target was 98.2% ^160^Gd enriched, with 1% ^158^Gd, 0.25% ^157^Gd, 0.36% ^156^Gd, 0.18% ^155^Gd, and 0.01% ^154^Gd. With 10 mg of [^160^Gd]Gd_2_O_3_, typically 7–10 GBq of ^161^Tb was produced. The material was dissolved in high purity 1 M HNO_3_. The ampule was rinsed with H_2_O and the activity was combined.

All resins were preequilibrated in 20% aq. MeOH for 1 h before use. TK212 and TK211 (1 mL each) columns were prepared and conditioned as described above. TK221 (30 µL) was packed to a 200 µL micropipette tip. A small piece of polyethylene frit was pushed to the narrow side of the tip, the TK221 resin was added, and another larger piece of frit was added on top. The column was washed with 300 µL of 0.75 M HNO_3_ For the semi-automated runs, the conditioning of the TK212 and TK211 columns was included in the automation sequence of the TRASIS, for the manual trial pre-equilibration was conducted manually. All flow rates were kept to 1 mL/min.

Unpurified ^161^Tb (50–110 MBq, 0.75 MBq/µL, 0.08 M HNO_3_) was diluted to 1 mL with 0.2 M HNO_3_ in a 1 mL centrifuge tube. The material was then loaded on to TRASIS All-in-one Mini module and separated as follows: TK212 was rinsed with 10 mL of 0.2 M HNO_3_ and this portion was collected for target recycling as it contains the bulk of the ^160^Gd. Next the ^161^Tb and ^161^Dy were eluted from the TK212 column with 10 mL 0.5 M HNO_3_, which was directly loaded onto the TK211 column. The TK211 column was rinsed with 35 mL 0.5 M HNO_3_ to further reduce the ^160^Gd content. Then 15 mL 0.75 M HNO_3_ was used to elute ^161^Tb off the TK211 column and leave the bulk of the ^161^Dy retained. This portion was directly loaded on to a TK221 column. At this point the automation ended and the following steps were performed manually. The TK221 column was rinsed with 150 µL 0.1 M HNO_3_ followed by 60 µL of 4 M HCl before finally eluting with 180–300 µL of 0.05 M HCl to obtain the final ^161^Tb product. The final elution was fractionated to ensure a more concentrated Tb product. The addition of the 4 M HCl rinse was done to remove the bulk of the nitric acid and allowed for a sharper elution of the final ^161^Tb product with 0.05 M HCl.

Three experiments at 50 MBq (activity recovery 90%, 0.164 MBq/µL at EOS), 50 MBq (activity recovery 71%, 0.198 MBq/µL at EOS) and 110 MBq (activity recovery 68%, 0.763 MBq/µL at EOS) were performed. ^161^Tb activity was determined by gamma spectroscopy, by dispensing a 5 µL aliquot of purified activity into a 20 mL scintillation vial for measuring.

For radionuclidic purity measurements, three samples (unpurified, HPIC purified, and small column purified ^161^Tb from the same batch, ~ 7.5 MBq each sample at EOS) were allowed to decay for 70 days and then re-measured. For small SPE column purified sample, the product from the 110 MBq purification was used. Each sample was diluted to 20 mL in a scintillation vial and counted for 15 h by a gamma spectrometer. The minimal detectable activities (10% confidence factor, 5% Bayesian confidence factor) are: ^46^Sc: 0.75 Bq, ^141^Ce: 3.6 Bq, ^152^Eu: 1.6 Bq, ^153^Gd: 2.3 Bq, ^154^Eu: 0.97 Bq, ^155^Eu: 2.0 Bq, ^156^Eu: 84 Bq, ^160^Tb: 2.0 Bq, ^161^Tb: 1.6 kBq; ^169^Yb: 5.0 Bq, ^192^Ir: 24 Bq.

With a separate shipment of unpurified ^161^Tb, purification was performed completely manually as outlined above. 190 MBq of unpurified ^161^Tb was successfully purified with an activity recovery 90% (1.08 MBq/µL at EOS).

#### ICP-MS analysis of ^161^Tb

Radioactive samples (~ 30 ppt) were prepared with the aid of gamma spectroscopy measurements. Samples were taken from final fractions of both small SPE column and the HPIC methods described earlier. In the same batch was run a series of 16 multielement standards, containing natural Gd, Tb, and Dy to generate the necessary calibration curves. Each sample and standard was measured in both He, and NH_3_ mass shift mode. ^159^Tb was measured in He mode using the ^159^Tb calibration curve for quantification, ^160^Gd was measured in NH_3_ mass shift mode (^160^Gd^+^→^160^GdNH^+^, M + 15), and quantified using ^160^Gd calibration curve. This was done to eliminate interference from ^160^Dy in the multielement standard. ^161^Tb was measured in NH_3_ mass shift mode (^161^Tb^+^→^161^TbNH^+^, M + 15) and quantified by comparing the resulting signal to that the ^159^Tb curve generated in the same tuning mode. ^161^Dy was measured by first determining the resultant counts of ^161^Tb in He then subtracting this number from the total counts observed at m/z 161, then ^161^Dy could be quantified by simply using the calibration curve generated for ^161^Dy in He tune mode.

#### Concentration dependant radiolabelling

100 kBq of ^161^Tb was buffered to pH 6 using 1 M pH 7 NH_4_OAc. The ultra-pure water and ligand were added to the reaction to achieve the desired final ligand concentration (total volume 10 µL). Reactions with crown were allowed to react for 30 min at room temperature (~ 20 °C) and reactions with DOTA were allowed to react for 30 min at 85 °C. Once the reactions were completed a portion (5 µL) of the reaction was spotted onto silica TLC plates with aluminum backing and the plates were allowed to develop in 50 mM pH 5.5 EDTA. Once the plates were fully developed the activity on the plates was scanned. Under these conditions, unchelated Tb^3+^ moves to the solvent front (R_f_ = 0.8–1.0), and the Tb-ligand complexes stay at the origin of the plate (R_f_ < 0.2).

#### Preparation of [^161^Tb]Tb-crown-αMSH for highest apparent molar activity experiments

Highest apparent molar activity of [^161^Tb]Tb-crown-αMSH was determined by mixing increasing amount of ^161^Tb in 0.05 M HCl (10–20 µL of 0.732 MBq/µL for HPIC purified ^161^Tb, 10–15 µL of 0.712 MBq/µL for small column purified ^161^Tb), NH_4_OAc buffer (1 M, pH 5–6, 2 µL) and crown-αMSH (10^–4^ M, 1 µL). The reactions were kept at 37 °C for 30 min. The RCC of the reactions was assessed after 30 min via iTLC SG plates and developing the plates with 50 mM pH 5.5 EDTA. Once the plates were fully developed the activity on the plates was scanned by radioTLC scanner. Under these conditions, unchelated Tb^3+^ moves to the solvent front (R_f_ = 0.8–1.0), and the Tb-ligand complexes stay at the origin of the plate (R_f_ < 0.2). Multiple trials were carried out with all reaction volumes kept to a minimum. The ratio of ^161^Tb activity (MBq) to the amount of crown-αMSH (nmol) was increased until the reaction was no longer able to produce RCC ≥ 99% as assessed by iTLC. The experiments were conducted 5 days after initial purification of ^161^Tb for both HPIC and small column purified products.

#### Biodistribution study

Male C57BL/6 J mice were inoculated with B16-F10 tumors using method previously reported at British Columbia Cancer Research Institute (Yang et al. 2020). Two to four days after inoculation, the mice were transferred to the UBC Centre of Comparative Medicine, where biodistribution studies were performed. Tumor size range from 0.28 to 0.76 g.

[^161^Tb]Tb-crown-αMSH was prepared by mixing ^161^Tb (15 µL 22.09 MBq HPIC purified ^161^Tb, or 15 µL 10.75 MBq small column purified ^161^Tb), NH_4_OAc buffer (5 µL, 1 M, pH 7) and crown-αMSH (10^–4^ M, 2.7 µL). Reactions were kept at 37 °C for 30 min. Molar activities were 39.8 MBq/nmol for small column purified and 81.8 MBq/nmol for HPIC purified [^161^Tb]Tb-crown-αMSH. The product was analyzed by radioTLC and radioHPLC and showed RCC > 97%. HPLC was performed using a Phenomenex Luna C18 reverse phase column (100 × 4.6 mm, 5 µm) with A: 0.1% TFA in water, B: 0.1% TFA in acetonitirle. With gradient 100% A→100% B in 15 min and flow rate at 1 mL/min, the retention time was 9.2 min. The product was diluted with injectable saline and used without purification.

For biodistribution studies, ~ 500 kBq [^161^Tb]Tb-crown-αMSH (range: 383–396 kBq 9.6–9.9 pmol small column purified) (range 647–655 kBq 7.9–8.0 pmol HPIC purified) was injected to each animal in the tail vein. After injection, the mice were allowed to move freely in their cages, and they were euthanized at 2 h post injection by CO_2_ asphyxiation under isoflurane anaesthesia. Blood was collected by cardiac puncture and a full biodistribution was performed. Organs were cleaned from blood, weighed, and the activity determined using a calibrated gamma counter (Packard Cobra II Auto-gamma counter, Perkin Elmer) using energy windows 35–60 keV. Counts and injection dose were decay corrected to the time of sacrifice and total organ weights were used for the calculation of injected dose per gram of tissue (%ID/g). Three animals were included in each group. %ID/g was expressed as average ± standard deviation, which was calculated by Microsoft Excel.

## Supplementary Information

**Additional file 1**. Supplementary file includes ICP-MS calibration curves (**Fig. S1.** for ^159^Tb under He mode, **Fig. S2.** for ^161^Dy under He mode, **Fig. S3.** for 160Gd(NH)^+^, **Fig. S4.** for ^159^Tb(NH)^+^, and **Fig. S5.** for ^159^TbNH(NH_3_)^+^), along with ICP-MS parameters for He tune mode (**Table S1**) and NH_3_ tune mode (**Table S2**), and biodistribution data (**Table S3**).

## Data Availability

The datasets generated or analysed during the current study are included in this article or in the Additional file 1.

## Abbreviations

PET: Positron emission tomography
SPECT: Single photon emission computed tomography
SPE: Solid phase extraction
HPIC: High performance ion chromatography
ICP-MS: Inductively coupled plasma mass spectrometry
GMP: Good manufacturing practice
MC1R: Melanocortin 1 receptor
RCC: Radiochemical conversion
