# Supplementary material for: A simple and automated method for 161Tb purification and ICP-MS analysis of 161Tb
Source: EJNMMI Radiopharm Chem. 2022 Dec 2;7:31. doi: 10.1186/s41181-022-00183-y (PMC9718904; doi:10.1186/s41181-022-00183-y)
Supplement: Supplementary file 1 — Additional file 1. Supplementary file includes ICP-MS calibration curves (Fig. S1. for 159Tb under He mode, Fig. S2. for 161Dy under He mode, Fig. S3. for 160Gd(NH)+, Fig. S4. for 159Tb(NH)+, and Fig. S5. for 159TbNH(NH3)+), along with ICP-MS parameters for He tune mode (Table S1) and NH3 tune mode (Table S2), and biodistribution data (Table S3). [file 41181_2022_183_MOESM1_ESM.pdf]

Supplementary Materials for

A simple and automated method for ^{161}Tb purification and real-time ICP-MS analysis

Scott W. McNeil[1], Michiel Van de Voorde[2], Chengcheng Zhang[3], Maarten Ooms[2], François Bénard[3], Valery Radchenko[1,4], Hua Yang[1,5*]

[1]Life Sciences Division, TRIUMF, 4004 Wesbrook Mall, Vancouver, BC, V6T 2A3, Canada

[2]Belgian Nuclear Research Centre: Studiecentrum voor Kernenergie, Boeretang 200, 2400 Mol, Belgium

[3]Department of Molecular Oncology, British Columbia Cancer Research, 675 West 10th Ave., Vancouver, BC, V5Z 1L3, Canada

[4]Department of Chemistry, University of British Columbia, 2036 Main Mall, Vancouver, BC, V6T 1Z1, Canada

[5]Department of Chemistry, Simon Fraser University, 8888 University Dr, Burnaby, BC V5A 1S6, Canada

* Corresponding author: Hua Yang, hyang@triumf.ca, Life Sciences Division, TRIUMF, Vancouver, Canada

Table of Contents

ICP-MS Calibration curves:.......... 2

ICP-MS Parameters: 4

Biodistribution data:.......... 7

ICP-MS Calibration curves:

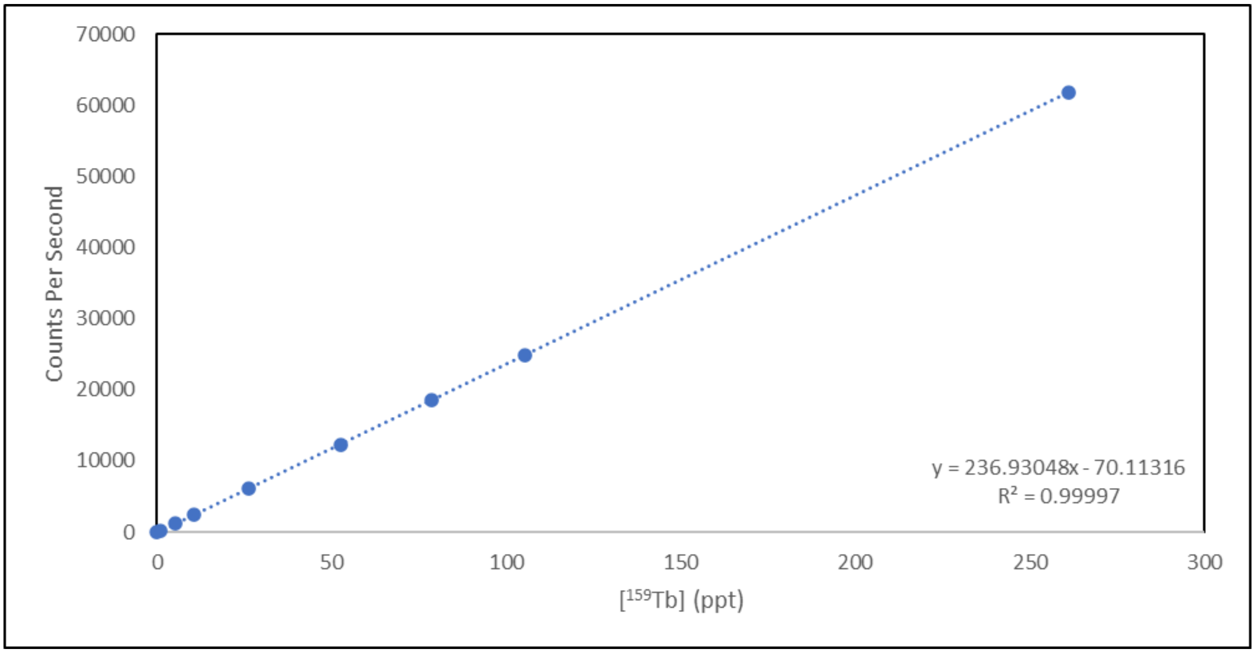

Fig. S1: He mode ^{159}Tb calibration curve

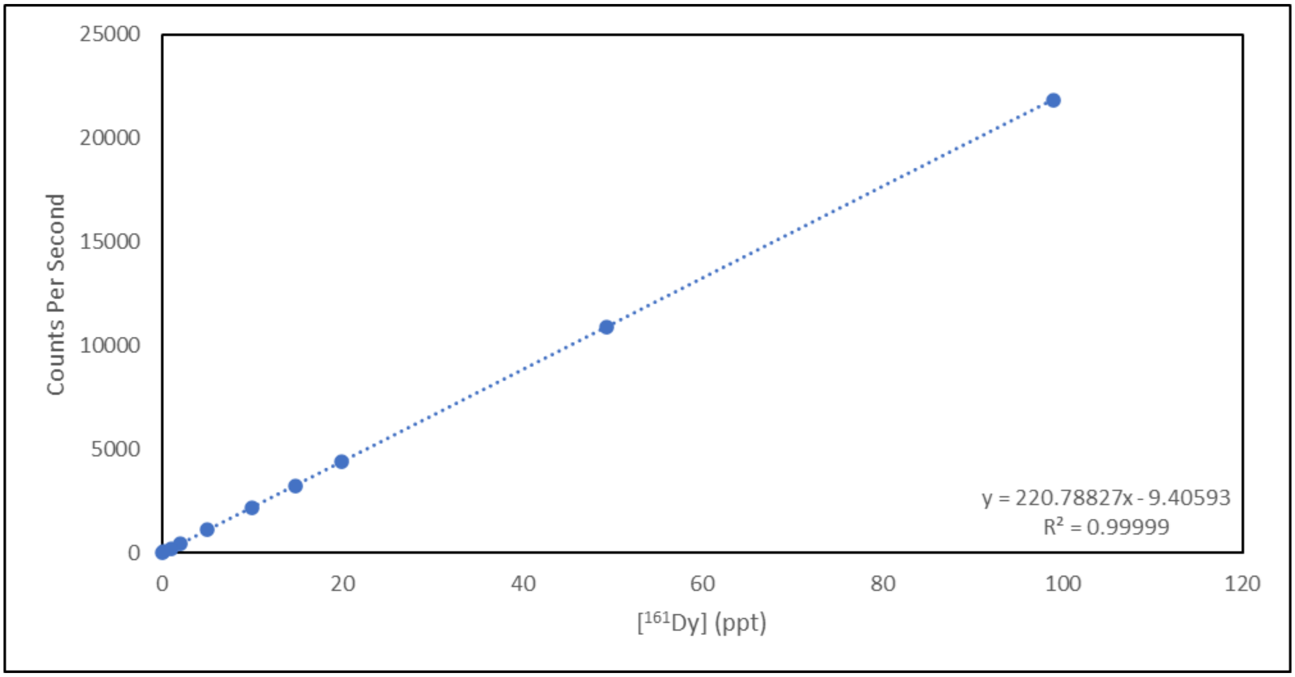

Fig. S2: He mode ^{161}Dy calibration curve

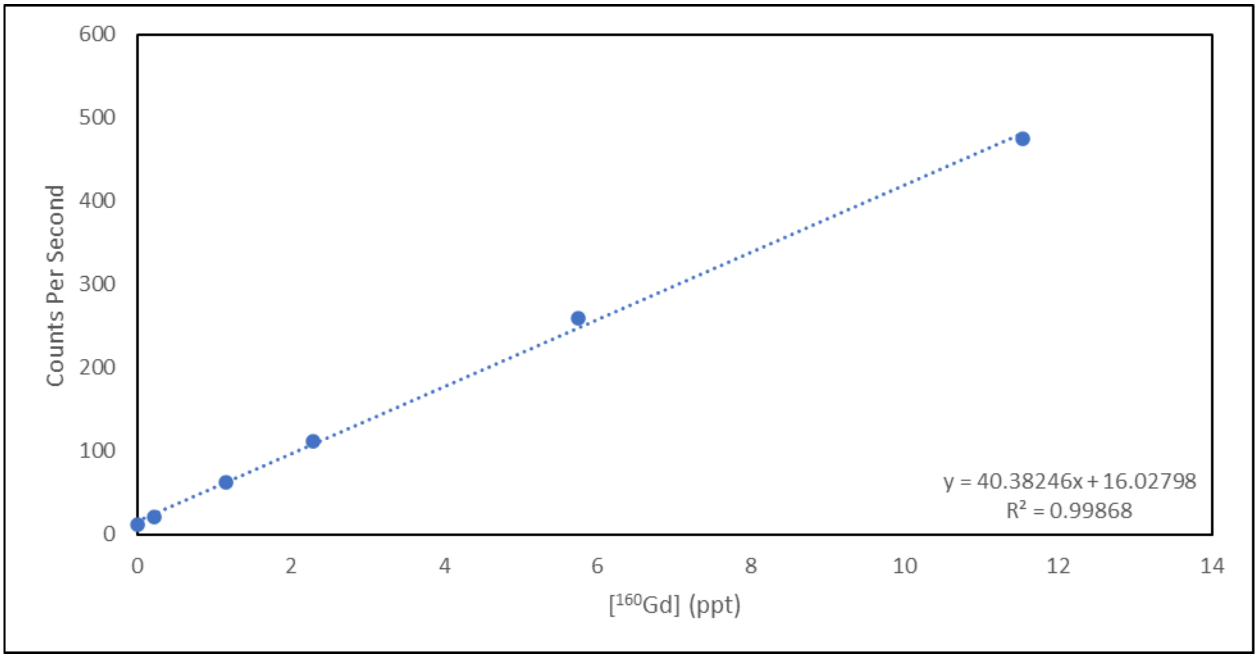

Fig. S3: NH_3 MS/MS mode $^{160}Gd^+ \rightarrow ^{160}GdNH^+$ calibration curve

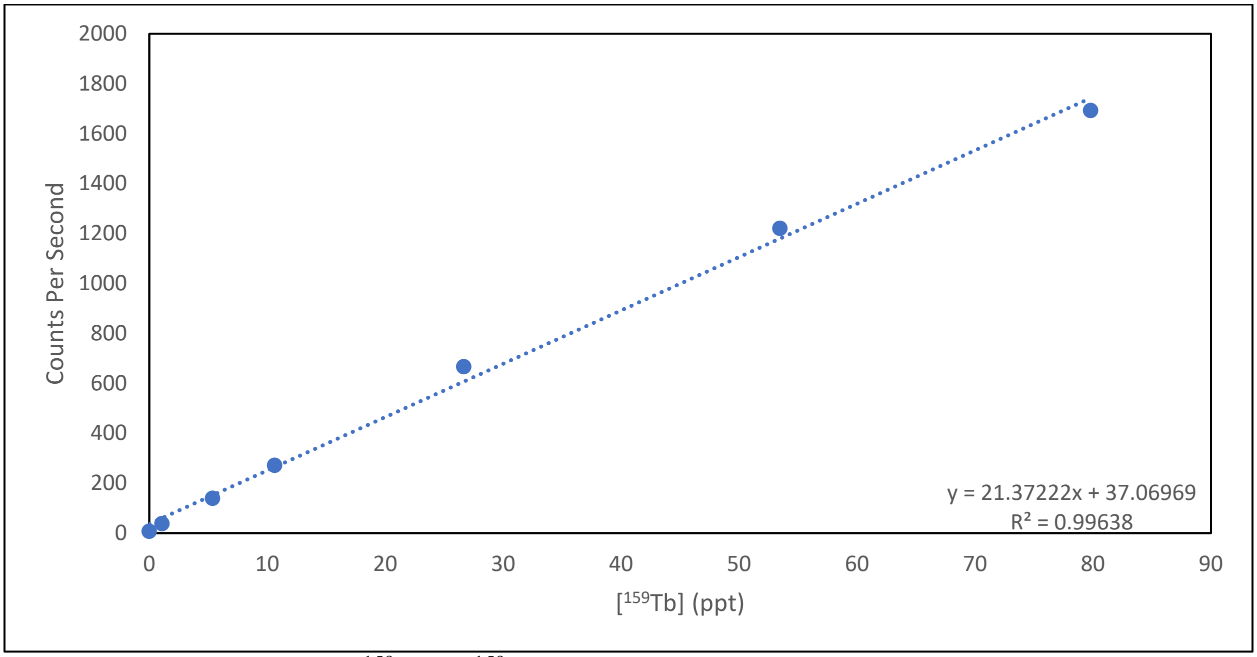

Fig S4: NH_3 MS/MS mode $^{159}Tb^+ \rightarrow ^{159}TbNH^+$ calibration curve

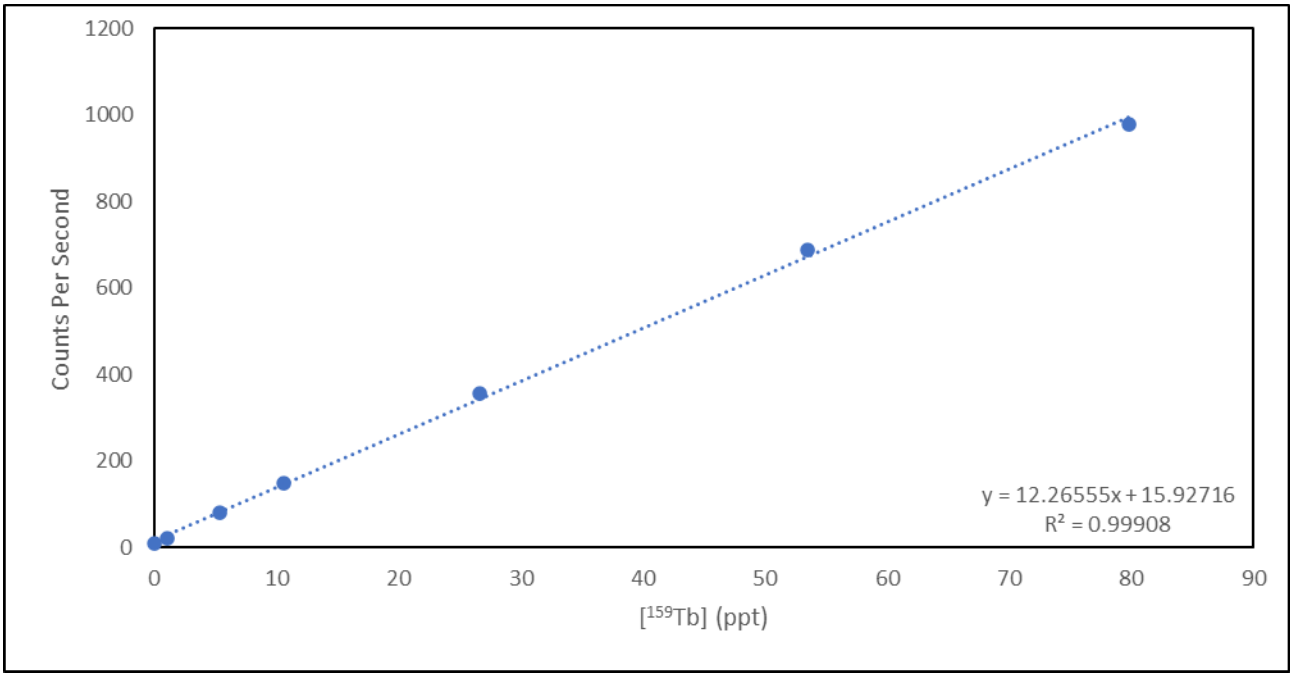

Fig. S5: NH_3 MS/MS mode $^{159}Tb^{+} \rightarrow {}^{159}TbNH(NH_3)^{+}$ calibration curve

ICP-MS Parameters:

Instrument: Agilent 8900 #100 ICP-MS QQQ

Table S1: ICP-MS parameters for He Tune mode

Parameter	Setting
**Scan Type**	Single Quad
**Plasma Mode**	Low Matrix
**RF Power**	1550 W
**RF Matching**	1.40V
**Sample Depth**	8.0 mm
**Nebulizer Gas**	1.05 L/min
**Option Gas**	0.0 %
**Nebulizer Pump**	0.10 rotations/s
**Spray Chamber Temperature**	2 °C
**Makeup Gas**	0.00 L/min
**Plasma Gas**	15.0 L/min
**Auxiliary Gas**	0.9 L/min
**Extract 1**	-9.6 V
**Extract 2**	-250.0 V
**Omega Bias**	-140 V
**Omega Lens**	11.0 V

**Q1 Entrance**	-50 V
**Q1 Exit**	0.0 V
**Cell Focus**	-2.0 V
**Cell Entrance**	-50 V
**Cell Exit**	-60 V
**Deflect**	-4.2 V
**Plate Bias**	-60 V
**Q1 Bias**	-7.0 V
**Q1 Prefilter Bias**	-9.5 V
**Q1 Postfilter Bias**	-8.0 V
**He Flow**	On
**He Flow Rate**	5.0 mL/min
**H2 Flow**	Off
**H2 Flow Rate**	0.0 mL/min
**NH3 Gas Flow**	Off
**NH3 Flow Rate**	0 %
**O2 Gas Flow**	Off
**O2 Flow Rate**	0 %
**Octupole Bias**	-18.0V
**Axial Acceleration**	1 V
**Octupole RF**	180 V
**Energy Discrimination**	3.0 V
**Wait time Offset**	0 ms

Table S2: ICP-MS parameters for NH_3 Tune mode

Parameter	Setting
**Scan Type**	MS/MS
**Plasma Mode**	Low Matrix
**RF Power**	1550 W
**RF Matching**	1.40V
**Sample Depth**	8.0 mm
**Nebulizer Gas**	1.05 L/min
**Option Gas**	0.0 %
**Nebulizer Pump**	0.10 rotations/s
**Spray Chamber Temperature**	2 ºC
**Makeup Gas**	0.00 L/min
**Plasma Gas**	15.0 L/min
**Auxiliary Gas**	0.9 L/min
**Extract 1**	-9.6 V
**Extract 2**	-250.0 V
**Omega Bias**	-140 V
**Omega Lens**	11.0 V
**Q1 Entrance**	-50 V
**Q1 Exit**	0.0 V

**Cell Focus**	-2.0 V
**Cell Entrance**	-50 V
**Cell Exit**	-60 V
**Deflect**	4.8 V
**Plate Bias**	-60 V
**Q1 Bias**	-4.0 V
**Q1 Prefilter Bias**	-9.0 V
**Q1 Postfilter Bias**	-10.0 V
**He Flow**	On
**He Flow Rate**	1.0 mL/min
**H2 Flow**	Off
**H2 Flow Rate**	0.0 mL/min
**NH3 Gas Flow**	On
**NH3 Flow Rate**	30 %
**O2 Gas Flow**	Off
**O2 Flow Rate**	0 %
**Octupole Bias**	-5.0 V
**Axial Acceleration**	0.5 V
**Octupole RF**	180 V
**Energy Discrimination**	-7.0 V
**Wait time Offset**	2 ms

Biodistribution data:

Table S3: Biodistribution of [^{161}Tb]Tb-crown-αMSH using ^{161}Tb from both HPIC and Small column purification method, n=3

	**Small Column**		**HPIC**	
**Organ**	%ID/g	Standard Deviation	%ID/g	Standard Deviation
**Blood**	0.16	0.08	0.25	0.06
**Urine**	114.88	97.83	128.99	56.44
**Feces**	0.35	0.50	0.25	0.07
**Tail**	0.58	0.13	0.77	0.41
**Brain**	0.02	0.00	0.02	0.00
**Muscle**	0.04	0.01	0.06	0.04
**Bone**	0.41	0.10	0.30	0.05
**Spleen**	0.19	0.03	0.21	0.02
**Pancreas**	0.06	0.01	0.08	0.02
**Stomach**	0.77	0.19	0.54	0.11
**Kidneys**	4.48	0.74	4.02	0.18
**Liver**	0.85	0.11	0.64	0.11
**Heart**	0.10	0.04	0.15	0.03
**Lungs**	0.32	0.18	0.32	0.03
**Small Int.**	0.40	0.09	0.41	0.22
**Large Int.**	0.31	0.05	0.33	0.13
**Tumour**	6.81	0.12	6.81	1.58
**Thyroids**	0.72	0.03	0.90	0.37
